# Introduction to Special Issue of Molecular Plant Pathology ‐ “Extracellular and intracellular perception of plant viruses”

**DOI:** 10.1111/mpp.12842

**Published:** 2019-08-29

**Authors:** Miguel A. Aranda, Kristiina Mäkinen, Jeanmarie Verchot

Miguel A. Aranda

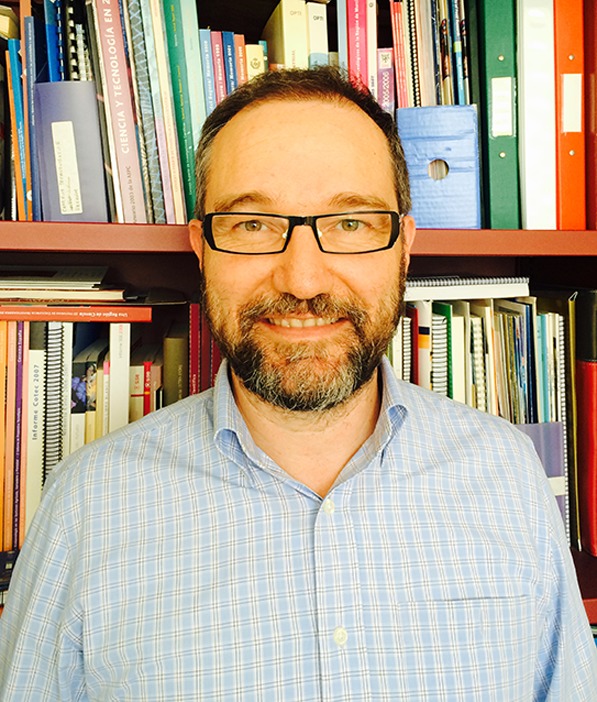

Kristiina Mäkinen

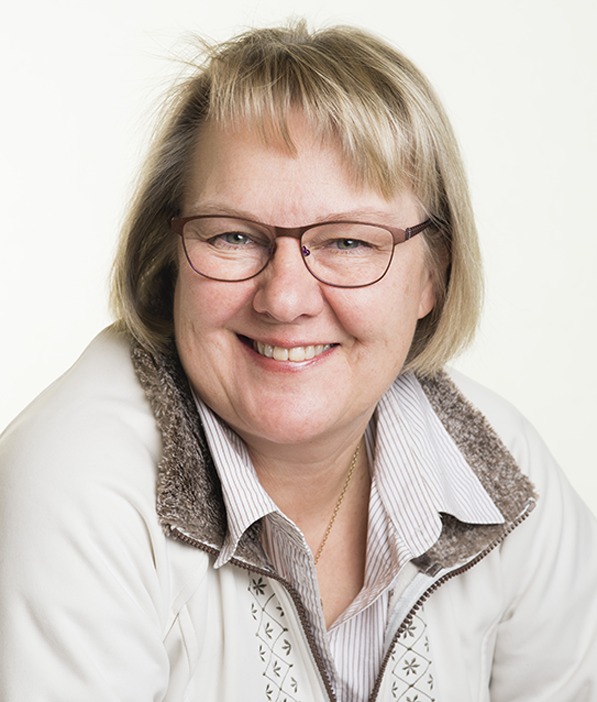

Jeanmarie Verchot

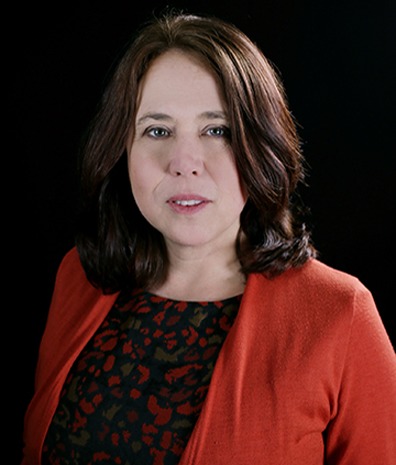

We are very pleased to present in this Special Issue of Molecular Plant Pathology a series of short reviews on the topic “Extracellular and intracellular perception of plant viruses”. The issue summarizes important breakthroughs in plant defence against viruses. Significant advances made in recent years provide new insights into the mechanisms of antiviral immunity after virus recognition by host cells. Plant viruses constitute an important cause of major crop losses worldwide and threaten food security. The advances in the field of plant–virus interactions will expand the potential strategies for breeding and engineering resistance in crops. The articles in this issue discuss the co‐evolving virulence and plant resistance strategies exemplifying the evolutionary arms race between viruses and their hosts.

For the past twenty years, the endogenous RNA silencing pathway acting to degrade viral RNAs, and intracellular perception of viral proteins by nucleotide‐binding leucine‐rich repeat (NLR) receptors leading to effector‐triggered immunity (ETI), have been seen as the most critical contributions to antiviral defence in plants. As discussed in the review entitled “Perspectives on intracellular perception of plant viruses” by Meier *et al*. in this issue, a considerable body of knowledge on how viruses are perceived by NLRs, and how NLRs are activated and use downstream signalling pathways to initiate defence responses, has developed during these years. In spite of that, our understanding of the underlying phenomena is still far from complete. After their concise but comprehensive review, the authors draw future lines for research, and point out structural analyses of NLRs complexed with their partners as one source of critical information to understand intracellular perception of plant virus and defences activation.

In a first layer of the plant immune system, extracellular perception of pathogen‐associated molecular patterns (PAMPs) leads to pathogen‐triggered immunity (PTI). Recent findings strongly suggest the existence of PTI‐like responses against viral infection. The newly uncovered layer of antiviral defence initiates at the plasma membrane and leads to a broad range of early responses to plant viruses. As discussed in “Molecular dialogues between viruses and receptor‐like kinases in plants” by Macho and Lozano‐Duran, activation of receptor‐like kinases (RLKs) on the cell surface during virus infection leads to activation of PTI‐related genes and multiple outcomes of plant's antiviral defence. Effects on the level of developmental rearrangements and cell‐to‐cell movement of RNAi are also possible. It is under debate whether plant viral proteins can directly act as PAMPs or whether the infection induces production of danger‐associated molecular patterns (DAMPs) for RLK activation. An increasing body of evidence is revealing that RKLs are targets of viral proteins. Teixeira *et al*. also discuss these aspects in their review entitled “Virus perception at the cell surface: revisiting the roles of receptor‐like kinases as viral pattern recognition receptors”. Importantly, they make a case of the NUCLEAR SHUTTLE PROTEIN (NSP)‐INTERACTING KINASE 1 (NIK1), an RLK identified in an interaction screening with NSP, which is a begomoviral protein. Activation of NIK1 leads to repression of ribosomal protein genes, suppression of global translation, including begomoviral mRNAs, and confers resistance to begomoviruses. Their data nicely fits in a model in which begomovirus‐derived nucleic acids could act as PAMPs to activate NIK1, and NSP acts as a viral effector that suppresses NIK1. Whether or not this model could be extended outside of begomoviruses is a matter of future studies.

Both RNA silencing and, as recently found, antiviral PTI, recognize double‐stranded (ds) RNAs as defence signals. During virus infection, replication and expression of viral genomes leads to accumulation of virus‐derived transcripts and dsRNAs. Since viruses are intracellular pathogens, we have well accepted that the cellular RNA silencing machinery recognizes viral RNAs. Antimicrobial PTI, however, involves plasma membrane‐localized pattern‐recognition receptors (PRRs). In the insightful review entitled “Perception of double‐stranded RNA in plant antiviral immunity”, Annette Niehl and Manfred Heinlein discuss several different scenarios on how perception of dsRNA by PRRs could happen during dsRNA‐mediated antiviral PTI.

The series of papers closes with a review on autophagy and plant viruses. During the recent years, autophagy has turned out to have an important role in plant virus infection and antiviral immunity as highlighted by Kushwaha, Hafren and Hoffius in their review entitled “Autophagy‐virus interplay in plants: from antiviral recognition to pro‐viral manipulation”. The authors discuss a wide variety of examples of autophagy in antiviral recognition and degradation, pro‐viral counterdefence and manipulation, disease‐related cell death, immunity‐related hypersensitive reaction and other viral functions.

The coming years will inevitably deepen our still limited knowledge on the mechanisms and functions of extracellular and intracellular recognition of viruses, and the contribution of autophagy in plant virus infection. We are sincerely convinced that this series of reviews can contribute to identify new important research avenues, particularly for young researchers joining the exciting field of plant virology.

